# Exercise-induced up-regulation of *MMP-1 *and *IL-8 *genes in endurance horses

**DOI:** 10.1186/1472-6793-9-12

**Published:** 2009-06-24

**Authors:** Katia Cappelli, Michela Felicetti, Stefano Capomaccio, Camillo Pieramati, Maurizio Silvestrelli, Andrea Verini-Supplizi

**Affiliations:** 1 Pathology, Diagnostic and Veterinary Clinic Department, University of Perugia, Via San Costanzo 4, 06126 Perugia, Italy

## Abstract

**Background:**

The stress response is a critical factor in the training of equine athletes; it is important for performance and for protection of the animal against physio-pathological disorders.

In this study, the molecular mechanisms involved in the response to acute and strenuous exercise were investigated using peripheral blood mononuclear cells (PBMCs).

**Results:**

Quantitative real-time PCR (qRT-PCR) was used to detect modifications in transcription levels of the genes for matrix metalloproteinase-1 (*MMP-1*) and interleukin 8 (*IL-8*), which were derived from previous genome-wide expression analysis. Significant up-regulation of these two genes was found in 10 horses that had completed a race of 90–120 km in a time-course experimental design.

**Conclusion:**

These results suggest that *MMP-1 *and *IL-8 *are both involved in the exercise-induced stress response, and this represents a starting point from which to understand the adaptive responses to this phenomenon.

## Background

It is widely accepted that moderate physical activity may have beneficial effects on the overall health of humans and other animals, while strenuous physical effort induces an inflammation-like state, whose grade depends on the type, intensity and duration of the activity [1,2]. The molecular mechanisms that underlie the cellular response to this phenomenon are still unclear; however, it is well known that it involves the coordinated action of multiple organ systems. The process begins with disruption of the homeostasis of the neuroendocrine-immune system, which alters the concentrations of neurotransmitters, hormones and cytokines. Exercise is followed by an intensity-dependent increase in sympathetic activity, which involves activation of the hypothalamus-pituitary axis. This leads to systemic secretion of catecholamines, which influence various immunological processes, including lymphocyte proliferation and differentiation, and production of cytokines [3,4]. Knowledge of the molecular mechanism of acute exercise-induced stress is a fundamental prerequisite for the planning of appropriate training schedules to improve the performance and maintain the welfare of the athlete.

Like a human marathon runner, the endurance horse undergoes extreme effort and can be considered a good model for the study of exercise-induced stress.

Given that exercise has been shown to be an important factor in the regulation of immune cells and their functions, and considering that stress evokes inflammatory reactions, peripheral blood mononuclear cells (PBMCs) are thought to be the best cell type in which to investigate the physiological changes associated with strenuous exercise [5]. Changes in the expression of a group of genes within the leukocytes may serve as surrogate markers for systemic or local modifications induced by exercise, and thus obviate the need for muscle biopsies [6].

The response of leukocytes to physical exercise, which involves the expression of multiple genes, is well known. Nevertheless, we are far from having a complete list of genes that are differentially expressed and, above all, far from a deep understanding of the regulatory mechanisms involved.

Analysis of leukocytes using microarrays has allowed the changes in immune function associated with exercise to be better understood [5,7-11]. Despite these interesting findings, variations in methodological conditions (microarray platforms, heterogeneous cellular populations, etc.) make the comparison of the results of array analysis difficult. For example, a maximum of only 3 to 11 concordant genes were found to overlap in four different microarray analyses of leukocytes after exercise [12]. Another technique that can be applied to this phenomenon is cDNA-AFLP [13]; this method allows novel genes to be identified without any previous sequence information. This is a robust and sensitive technology with low start-up costs that is used for discovery of genes on the basis of fragment detection [14].

This technique of genome-wide expression analysis has been used to study exercise-induced stress in endurance horses. It allowed us to identify, among a large set of genes, two transcript-derived fragments that were differentially expressed: one (FG341843) encoding metalloproteinase 1 (*MMP-1*) and one (CO508730) encoding interleukin 8 (*IL-8*).

Metalloproteinases (MMPs) and chemokines, which are induced by the inflammatory response, are involved in the redistribution of lymphocytes between lymphoid and non-lymphoid organs and the mobilization of haematopoietic progenitor cells (HPCs) from bone marrow. These processes have been shown recently to be related to exercise-induced stress [5,15,16]. The major chemokine that is able to increase the number of mobilized HPCs is IL-8, and this chemokine also stimulates the production of MMPs. Matrix metalloproteinase-1 is involved in the enhanced peripheral invasion and migration into tissues of natural killer (NK) cells in humans. Production of MMP-1 from stimulated NK cells plays a role in the facilitation of lymphocyte trafficking, and in the accumulation of lymphocytes in tissues during physiological and pathological processes [17].

The aim of this study was to investigate the expression of *IL-8 *and *MMP-1*, which had been identified with a gene discovery technique [18], in equine athletes. In addition, with the use of data from previously published reports, we wished to determine the possible role of these genes in acute exercise-induced stress.

To achieve this goal, we used quantitative real-time PCR (qRT-PCR), which is the technique of choice to detect modifications in the level of transcription of specific genes in a reliable and reproducible manner.

## Results

The raw cycle threshold values (Cts) detected during qRT-PCR experiments by MxPro software 3.21 (Stratagene) were analysed using the software GenEx Pro [19]. This allowed the results of the real-time PCR to be analysed using two reference genes (*SDHA*, *HPRT*), with which the expression levels of the genes of interest were compared. This approach produces more consistent data in comparison with those obtained using only one reference gene [20,19].

**Figure 1:**
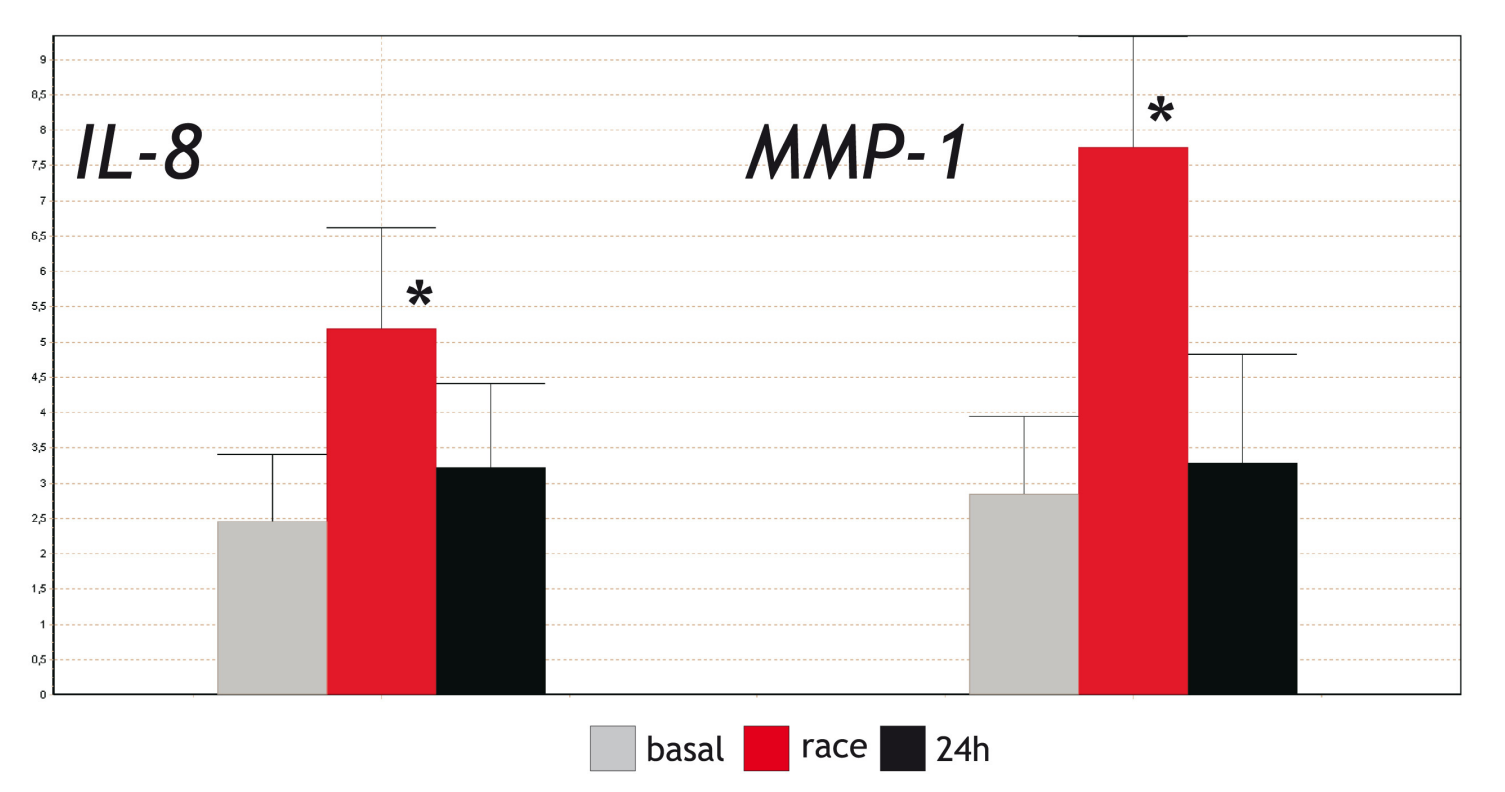
**Relative expression bar chart**. Bar chart of expression of *IL-8 *and *MMP-1 *in 10 endurance horses during a time course experiment (basal, race, 24 h). Error bars indicate the 95% confidence interval. Relative expression values are on a logarithmic base 2 scale. Asterisks indicate significance at *P *< 0.001.

During the pre-processing phase, data were corrected for the efficiency of the PCR (Table 1) and were averaged over the three technical repeats (considering only results that showed a standard deviation less than 0.2 within the triplicate). The selected reference genes *HPRT *and *SDHA *were used subsequently to normalize the Ct values, and the relative quantities were calculated on the basis of the maximum Ct value. Given our interest in the changes in the expression of genes between samples, the quantities were finally converted to a logarithmic scale using a log base 2 conversion. Changes in the relative expression of the genes encoding IL-8 and MMP-1 were checked for statistical significance using a non-linear mixed-effects model for repeated data using the *nlme *library from R [21], using version 2.7.2 of the software R. The resulting bar chart (Figure 1) shows an up-regulation (2.74 × for *IL-8*, *P *< 0.001; 5 × for *MMP-1*, *P *< 0.001 – log base 2 induction) immediately after the race, and a non-significant difference between *basal *and *24 hours *values (*IL-8 P *= 0.1464, *MMP-1 P *= 0.3689). These results demonstrate a significant up-regulation of *MMP-1 *and *IL-8 *induced by exercise in the PBMC of endurance horse in an *in vivo *system with a consistent number of animals.

**Table 1:** Real-time PCR primer pairs and assay conditions.

**Gene**	**Accession number**	**Primers (forward, reverse)**	**Amplicon length, bp**	**Efficiency of assay, %**	***r*^2^**
*MMP-1*	NM_001081847	CTGCTGCTGCTGCCACTC TCTCCATCACTCTTCAGGTTGTAG	131	100.8	1.000
*IL-8*	NM_001083951	CTGGCTGTGGCTCTCTTG CAGTTTGGGATTGAAAGGTTTG	132	97.8	0.999
*HPRT*	AY372182	AATTATGGACAGGACTGAACGG ATAATCCAGCAGGTCAGCAAAG	121	93.9	1.000
*SDHA*	DQ402987	GAGGAATGGTCTGGAATACTG GCCTCTGCTCCATAAATCG	91	96.0	0.999

In addition, the Pearson's correlation coefficient calculated between the level of expression of *IL-8 *and that of *MMP-1 *(ρ = 0.74) showed a strong relationship between the two genes.

## Discussion

In the present study, transcripts of *MMP-1 *and *IL-8 *were found to be strongly up-regulated during an equine endurance exercise and to be down-regulated 24 hours after the effort, which suggests a role for these genes in exercise-induced stress.

Matrix metalloproteinase-1 is a member of a large family of calcium-dependent zinc-containing endopeptidases, MMPs, which are responsible for tissue remodelling and degradation of the extracellular matrix (ECM) [22,23]. The expression of *MMP-1 *is stimulated at the transcriptional level by growth factors and cytokines [24,25].

The MMPs are secreted by connective tissues and by pro-inflammatory cells, including fibroblasts, osteoblasts, endothelial cells, macrophages, neutrophils and lymphocytes [23].

Interleukin 8 is a prototypical chemokine, and its most remarkable property is the wide variation in its level of expression in response to cellular stress. IL-8 is known to be produced by monocytes and macrophages, as well as by most tissues, and its secretion is induced by a number of stimuli, including pro-inflammatory cytokines [26,27]. The plasma concentration of IL-8 increases in response to strenuous exercise that is comprised of both eccentric and concentric components, such as running, and the regulation of *IL-8 *production occurs mainly at the level of transcription [28].

An attractive hypothesis concerning the role of *MMP-1 *and *IL-8 *in the response of PBMCs to physical stress is their involvement in lymphocyte trafficking and in the recruitment of progenitor stem cells from bone marrow.

Recent studies have shown that redistribution of leukocytes is a fundamental regulatory mechanism of the haematopoietic system that alters lymphocyte counts during exercise [15], as was observed in our samples obtained immediately after the race (data not shown). The ability of immune cells to migrate appears to be closely regulated by molecules such as chemokines and proteinases, and is mediated through receptors of the integrin family [29]. Cytokines stimulate the expression of multiple MMPs from lymphocytes. Recent studies suggest that MMP-1, produced by chemokine-stimulated NK cells, could play a role in the traversal of tissues by promoting the degradation of matrix collagens as well as by processing chemokines. Although the exact mechanism by which lymphocytes invade tissues is not completely understood, this migration seems to be regulated by distinct pathways that involve mitogen-activated protein kinases (MAPKs). Goda and colleagues [17] demonstrated that MMP-1 is produced by chemokine-stimulated NK cells and that it associates with α_2_β_1 _integrin, which suggests that integrins play a role in the immobilization of MMP-1 on the cell surface. The binding of MMPs to integrins would be a critical step for promoting lymphocyte migration, given that the disruption of this association decreases the migratory ability of cells [30]. It is also known that integrins are involved in the signalling pathway of the Rho family of GTPases that has been associated recently with modulation of the expression of *IL-8 *and *MMP-1*. When one of these key GTPases, Cdc42, is silenced, over-expression of *IL-8 *and *MMP-1 *is induced by inhibition of the ERK1/2 pathway [29].

In addition to the redistribution of lymphocytes, exercise is accompanied by the recruitment of stem cells and progenitor cells from the bone marrow [16]. Cytokines can increase the number of mobilized haematopoietic progenitor cells (HPCs) by releasing proteinases, and it has been demonstrated that IL-8 is the chemokine that shows the most rapid mobilization of HPCs in monkeys and mice [31]. IL-8 is also chemotactic for neutrophils and induces the release of metalloproteinases. The most commonly accepted hypothesis is that MMP-9, secreted by neutrophils, is required for the recruitment of HPCs [32]. However, unexpectedly, mice deficient in *MMP-9 *exhibited normal mobilization of HPCs, which suggests the existence of redundant pathways that compensate for the lack of this protease [33]. Our results, in addition to these previous findings, allow us to hypothesize that MMP-1 is part of this redundant pathway in PBMCs, and that it plays an important role in the mobilization of HCPs. This hypothesis is also supported by the over-expression of *IL-8 *that was observed in the 10 horses used in this work; IL-8 is considered to be a key chemokine in the mobilization of HCPs [31].

Moreover, our data confirm that there is a strong correlation between the up-regulation of *MMP-1 *and that of *IL-8*. The reason for the co-expression of *MMP-1 *and *IL-8 *in response to various stimuli, such as exercise-induced stress, could be found in their shared pathways and interactions with messenger molecules that are involved in the inflammatory and immune response.

## Conclusion

A significant co-induction of the *MMP-1 *and *IL-8 *genes was found, using a robust qRT-PCR approach, to occur immediately after strenuous exercise in PBMCs from 10 endurance horses. The quantitative method used in this study underlines the results obtained with genome-wide expression analysis, which was used to explore the *in vivo *stress system. Even if the induction of mRNA is not ever followed by an increase in the related protein and its activity, these results, together with evidence from the literature, suggest that IL-8 and MMP-1 are involved in the adaptation to exercise-induced stress and that they may have a role in lymphocyte trafficking and in the recruitment of progenitor stem cells from the bone marrow. Further evidence, such as proteomic data and the results of studies of cell mobilization tracking, will be required to confirm our findings.

## Methods

### Blood collection, RNA extraction and cDNA synthesis

Blood samples were taken from the jugular veins of horses chosen from the high-level participants in national and international endurance races (90–120 km). The samples were obtained at three different time points: before (*basal*), at the end of the race (*race*), and 24 hours after the race (*24 h*). Only horses that passed the FISE (Italian Equestrian Sports Federation) compulsory medical checks (pre, during and post-race) were considered suitable for this study. Ten endurance horses, comparable for training type and intensity, were recruited. The medical checks consisted of an examination of cardio-respiratory function, physical integrity, and metabolic condition, together with collection of a blood sample for a complete blood count.

The animals involved in this study were treated following standard procedures to ensure animal welfare, in agreement with the horse owners, team veterinarian and the official veterinary commission.

Immediately after collection of blood, peripheral blood mononuclear cells (PBMCs) were isolated by the Ficoll-Hypaque method (GE Healthcare, Pollards Wood, UK). Total RNA was extracted using the Aurum Total RNA Fatty and Fibrous Tissue kit (Bio-Rad, Hercules, CA, USA) according to the manufacturer's instructions. Genomic DNA was eliminated by a DNase treatment supplied with the kit. The extracted RNA was quantified using the Quant-It RNA assay (Invitrogen, Dorset, UK) in a VersaFluor fluorometer (Bio-Rad) and checked for integrity by denaturing agarose gel electrophoresis with ethidium bromide staining. Successful removal of DNA contaminants was tested by determining the absence of PCR amplification of the *MC1R *gene (GenBank accession number X98012, primers from Rieder and colleagues) [34]. Total RNA (1 μg) was retro-transcribed using random examers and Superscript III Reverse Transcriptase (Invitrogen) according to the manufacturer's specifications. A PCR for the *Equus caballus *β-actin gene (Forward 5'-GAGCAAGAGGGGCATCCTGA-3', Reverse 5'-GGTCATCTTCTCGCGGTTGG-3', Gene ID: 100033878) was performed on each sample of cDNA to check for successful retro-transcription.

### Reference gene selection and primer design

The genes encoding succinate dehydrogenase complex subunit 1 (SDHA) and hypoxanthine phosphoribosyl transferase (HPRT), which were the best house-keeping genes selected from a group of nine potential genes, were utilized to estimate gene expression accurately during exercise-induced stress in horses [35].

The primers were designed based on available sequences (Table 1) using Primer3 software [36]. Mfold [37] was used to check the chosen sequences to avoid designing primers in the region of the template secondary structure, and the primers were located, when possible, on different exons or at the exon-exon junction. The amplicon lengths ranged from 68 to 138 bp, to ensure optimal polymerization and efficiency. The specificity of target amplification was confirmed by sequencing.

For each primer pair, a preliminary real-time assay was performed to evaluate the amplification of non-specific products or primer dimer artefacts (no double peak in melt curve analysis). Efficiency was determined as follows: for each assay, a standard curve was generated by using 4-fold serial dilutions of pooled cDNAs. As showed in Table 1, linear correlation coefficients (r^2^) varied from 0.999 to 1.000 and PCR efficiencies (e) ranged between 93.9 and 100.8%.

### Real-time quantitative PCR

Five microliters of cDNA template (previously diluted 1:10) were added to the master mix FastStart SYBR Green Master (Roche Applied Science, Penzberg, Germany) with the ROX fluorochrome internal check. The PCR was performed in a volume of 25 μl on a MX3000P instrument (Stratagene, La Jolla, CA, USA). The PCR conditions were the same for all primer pairs: initial denaturation at 95°C for 10 min, followed by 40 cycles of denaturation at 95°C for 30 sec, annealing at 58°C for 30 sec and extension at 72°C for 30 sec. Fluorescence data were collected at the end of the extension step. Following cycling, the melting curve was determined in the range 58°-95°C, with a temperature increment of 0.01°C/sec. Each reaction was run in triplicate with appropriate negative controls.

Raw Ct from the MX3000P instrument were exported to a common exchange data file and analysed using GeneEx Pro [19] in order to correct the data against the two housekeeping genes and to calculate the Pearson correlation coefficient between the expression of *IL-8 *and that of *MMP-1*. The software R [21] was used to perform analysis of variance (ANOVA) with a non-linear mixed-effects model.

## Authors' contributions

KC drafted the manuscript and performed all experiments with MF. SC helped in drafting the manuscript and performed data analysis with CP's supervision. MS conceived the project, and AVS supervised and coordinated the project and participated in writing the manuscript. All authors read and approved the final manuscript.
